# Evaluation of Antioxidant Potential of “Maltese Mushroom” (*Cynomorium coccineum*) by Means of Multiple Chemical and Biological Assays

**DOI:** 10.3390/nu5010149

**Published:** 2013-01-11

**Authors:** Paolo Zucca, Antonella Rosa, Carlo I. G. Tuberoso, Alessandra Piras, Andrea C. Rinaldi, Enrico Sanjust, Maria A. Dessì, Antonio Rescigno

**Affiliations:** 1 Department of Biomedical Sciences, University of Cagliari, Monserrato 09042, Italy; E-Mails: pzucca@unica.it (P.Z.); anrosa@unica.it (A.R.); rinaldi@unica.it (A.C.R.); sanjust@unica.it (E.S.); dessima@unica.it (M.A.D.); 2 Consorzio UNO, Consortium University of Oristano, Oristano 09170, Italy; 3 Department of Life and Environmental Sciences, University of Cagliari, Cagliari 09124, Italy; E-Mail: tuberoso@unica.it; 4 Department of Chemical and Geological Sciences, University of Cagliari, Monserrato 09042, Italy; E-Mail: apiras@unica.it

**Keywords:** plant-based foods, antioxidant, nutraceuticals, *Cynomorium coccineum*, fungus melitensis, Maltese mushroom

## Abstract

*Cynomorium coccineum* is an edible, non-photosynthetic plant widespread along the coasts of the Mediterranean Sea. The medicinal properties of Maltese mushroom—one of the oldest vernacular names used to identify this species—have been kept in high regard since ancient times to the present day. We evaluated the antioxidant potential of fresh specimens of *C. coccineum* picked in Sardinia, Italy. Both aqueous and methanolic extracts were tested by using multiple assay systems (DPPH, FRAP, TEAC, ORAC-PYR). Total phenolics and flavonoids were also determined. Gallic acid and cyanidin 3-*O*-glucoside were identified as the main constituents and measured. Both extracts showed antioxidant capacities; ORAC-PYR assay gave the highest antioxidant value in both cases. The methanolic extract was further investigated with *in vitro* biological models of lipid oxidation; it showed a significant activity in preventing cholesterol degradation and exerted protection against Cu^2+^-mediated degradation of the liposomal unsaturated fatty acids. Results of the present study demonstrate that the extracts of *C. coccineum* show a significant total antioxidant power and also exert an *in vitro* protective effect in different bio-assays of oxidative stress. Therefore, Maltese mushroom can be considered a valuable source of antioxidants and phytochemicals useful in the preparation of nutraceuticals and functional foods.

## 1. Introduction

A large number of edible and/or medicinal plants contain chemical compounds that exhibit antioxidant properties [1,2]. Many studies have been performed on some of these plants which have provided positive results for the development of natural antioxidants to be used for the preparation of foods fortified with health-promoting additives, nutraceuticals endowed with health and medical benefits, and cosmetics [3]. Therefore, the study of new sources of natural antioxidants represents a useful and interesting challenge. 

**Figure 1:**
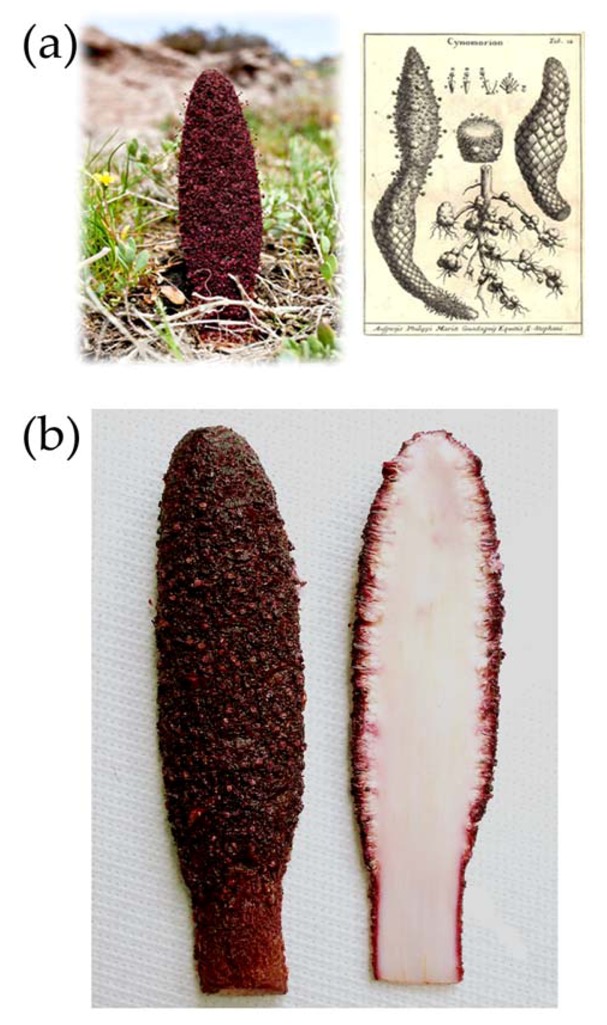
(**a**) Left, *Cynomorium coccineum* (Maltese mushroom), growing in a coastal area at Portoscuso, south-western Sardinia, Italy. The above ground height of the specimen was approx 20 cm; Right: A classic view of “fungus”: “Cynomorion” from *Nova Plantarum Genera*, P.A. Micheli (1729). (**b**) Slices of a specimen that show the comparison between the outside colored and the inner portion.

*Cynomorium coccineum* L. is a non-photosynthetic plant of the monogeneric angiosperm family Cynomoriaceae, spread over a large area of the Mediterranean basin. This species is found in the southern part of Italy (Sardinia, Sicily and Basilicata) and Spain, on the islands of Corsica, Malta and Crete, and from the North African coast to West Africa. Its range extends further to the Arabian Peninsula [4]. The plant is perennial and displays a very distinctive appearance and color (Figure 1); it is holoparasitic, often associated with coastal environments and saline soils, where it grows on the roots of salt-tolerant plants such as *Atriplex* and *Inula* [4].

The popular name “mushroom”, despite being a plant, is probably due to its appearance, the lack of chlorophyll and to the fact that it develops underground for most of the year. The plant is known under several vernacular names depending on the country in which it is present, the most common being Maltese mushroom, fungus melitensis, champignon or éponge de Malt, fungo di Malta, and, in Arabic countries, tarthuth [5]. The therapeutic properties of Maltese mushroom have been in high regard throughout the centuries. In the 16th century, the physicians of the Knights of Malta medieval order began using the plant in their treatments. On Malta, the “fungus” was appreciated as an aid for dysentery that plagued the military guards [5]. The value of *C. coccineum* as a medicinal remedy is, however, recorded in many other cultures, especially as concerns the use of the plant as antihaemorrhoidal, spermatogenesis stimulating agent, aphrodisiac, tonic, antivomitive, and hypotensive [5]. Some of these effects have been confirmed in studies in animal models [6,7]. The use of the “fungus” as a food, especially during periods of famine, is also reported [4] and may be due to its relatively high content of oils rich in essential fatty acids which helped people to survive when standard foods were insufficient. Recently, the composition and lipid profile of fixed oil from dried stems of *C. coccineum* has been investigated [8]. The latter study showed that the fixed oil extracted from this plant may be considered an important natural source of essential fatty acids.

The present work was aimed at evaluating the antioxidant properties of both aqueous and methanolic extracts of Maltese mushroom by means of various antioxidant assays.

For this purpose, both electron-transfer-based (ET) and hydrogen-atom-transfer-based (HAT) methods were included in this study, since significant differences between these two approaches have been outlined [9,10]. The biological profile of the methanolic extract obtained from *C. coccineum* was further investigated using *in vitro* models of lipid oxidation, during the thermal solvent-free oxidation of cholesterol and the copper-induced oxidation of liposomes. As the biological and chemical diversity of medicinal plants depends, among other variables, on factors such as geographic areas of growth, climatic conditions and genetic modifications we carried out this study only on *C. coccineum* plants growing in the island of Sardinia (Italy). 

## 2. Results and Discussion

### 2.1. Total Antioxidant Power and Phenolics Content

Both aqueous and methanolic extracts from *C. coccineum* were evaluated for their content of phenolics and flavonoids, and for their total antioxidant capacity. The selected ET-based methods were the DPPH assay, ABTS-based assay in order to measure the TEAC (Trolox Equivalents Antioxidant Capacity), and the FRAP method. The selected HAT-based method was the ORAC assay based on pyrogallol red. The full results are summarized in Table 1. Both extracts showed significant antioxidant capacities, with a few differences between them. ORAC-PYR assay gave in both cases the highest antioxidant capacity value, in accordance with the fact that this method is the only HAT-based, being thus able to evaluate overall antioxidant capacity, while FRAP, TEAC, and DPPH only evaluate the presence of reducing compounds (they are in fact electron-transfer based methods). This is in agreement with previously reported studies about the risk that ET-based assays may underestimate total antioxidant capacity [11]. Rached *et al.* (2010) have measured the antioxidant potential of specimens of *C. coccineum* grown in Algeria, using two complementary assays, namely inhibition of DPPH radical and β-carotene bleaching [12]. Other authors have studied the antioxidant activity in specimens of *C. songaricum*, an Asian species of the genus *Cynomorium*, also widely used in traditional Chinese medicine and known as “suoyang” [13,14].

**Table 1:** Total antioxidant capacity of aqueous and methanolic extracts from *C. coccineum*.

Assay	Methanol extract	Water extract
ORAC-PYR (mM TE/g)	0.91 ± 0.04	1.18 ± 0.06
DPPH (mM TE/g)	0.52 ± 0.01	0.50 ± 0.2
DPPH (IC50 μg/mL)	54.20 ± 2.1	51.6 ± 3.2
TEAC (mM TE/g)	0.89 ± 0.05	0.99 ± 0.11
TEAC (IC50 mg/mL)	0.91 ± 0.08	0.89 ± 0.04
FRAP (mM TE/g)	0.58 ± 0.02	0.50 ± 0.01
FRAP (mmol FeII/g)	1.35 ± 0.04	1.10 ± 0.03
Total phenolics (mM GAE/g)	1.02 ± 0.03	0.64 ± 0.02
Total flavonoids (mM CE/g)	0.139 ± 0.002	0.128 ± 0.003

Values are means ± SD; *n* =6*.*

When data reported in these studies are compared to what we found for the Maltese mushroom grown in Sardinia the results are not similar. In fact, the DPPH-based IC_50_ values of our extracts are significantly higher, showing a lower antioxidant capacity than Algerian *C. coccineum* and *C. songaricum *(4.1 μg/mL and 13.5 μg/mL for Algerian *C. coccineum *ethanolic and water extracts, and about 35 μg/mL for *C. songaricum*) [12,13]. On the contrary, the antioxidant capacity measured with FRAP assay appeared to be higher for our specimens (0.051 and 0.167 mmol Fe^II^/g for *C. songaricum *methanolic and water extracts respectively [14]). These conflicting data can be explained by the significant chemical and methodological differences between the two assays. Accordingly, for the full evaluation of the antioxidant capacity of any extracts, the use of a variety of assays is usually recommended [10,15]. The total content of phenolics was similar when comparing methanolic and aqueous extracts, and lower if compared with the content reported in other *Cynomorium* studies [12,14]. Among the total phenolics, the content of flavonoids represents a significant part, and this value for both extracts showed no significant differences if compared with the total flavonoids content observed in *C. coccineum* specimens picked in Algeria [12]. 

HPLC investigation of the fresh Maltese mushroom extracts revealed that the SFE extract had a chromatographic profile similar to that obtained for the methanol-water extraction (not shown). The chromatograms of both extracts showed a peak, identified as gallic acid, and two zones at about 32–33 and 37–39 min retention time of unidentified peaks. The UV-Vis spectra (200–600 nm) of these peaks revealed similarities with procyanidins and gallic acid derivatives, suggesting that they can be procyanidin oligomers. In the methanol-water extract of whole *C. coccineum* extract, the anthocyan cyanidin 3-*O*-glucoside was also detected. This is in agreement with that reported by Harborne *et al.* [16] who found the presence of cyanidin 3-*O*-glucoside in the red-brown inflorescences of *C. coccineum* where, like other anthocyans, it plays the role of attracting insect pollinators. Such investigation [16] reported that this is the only anthocyan of this plant, but in our extracts another five anthocyans were detected and quantified (Table 2). Interestingly, the external layer of *C. coccineum* is the richest in anthocyans, which are responsible for the intense plant color (Figure 1). The presence of considerable amounts of gallic acid and cyanidin 3-*O*-glucoside in the Maltese mushroom, when compared with other sources [17], is worthy of note. In fact, these are plant polyphenolic compounds naturally present in the human diet. Their use in dietary supplementation is well documented [18] as it can improve the antioxidative potential and nutritional and functional qualities of several foods [19], and is known to reduce the risk of disease [20,21].

**Table 2:** Phenolic composition of *C. coccineum* extracts (mg/g *).

Extract	Gallic acid	Cyanidin 3-*O*-glucoside	Other anthocyans ^#^
SFE CO_2_, whole plant	1.184 ± 0.079	0.009 ± 0.002	nd
Solvent, whole plant	4.573 ± 0.226	3.134 ± 0.071	0.042 ± 0.004
Solvent, external layer	3.413 ± 0.135	11.892 ± 0.676	0.507 ± 0.049
Solvent, peeled plant	2.894 ± 0.031	0.028 ± 0.002	tr

Values are means ± SD; *n* = 6; * Data are referred to g of dry extract; ^#^ Dosed as cyanidin 3-*O*-glucoside; nd = Not detected (<LOD); tr = Traces (<LOQ).

Taken together, these data demonstrate that extracts of *C. coccineum* show a significant total antioxidant potential, and that flavonoids reasonably give a contribution to the overall antioxidant capacity. This is quite interesting, since flavonoids seem to be involved in the anti-carcinogenic and anti-mutagenic activities of plant extracts [22]. 

### 2.2. Antioxidant Power in Models of Lipid Oxidation

To better assess the antioxidant activity observed with *in vitro* chemical assays, the methanolic extract obtained from *C. coccineum *was subsequently tested for its protective effect in a biochemical assay of oxidative stress, namely the thermal (140 °C), solvent-free degradation of cholesterol for 1 h or 2 h. The methanolic extract was chosen as the richest in total phenolics (Table 1), and also because, in general, methanolic extracts are more active when studying lipid models. Our model of lipid oxidation has been widely used to assess the antioxidant properties of extracts and natural compounds [23,24]. The consumption of cholesterol and its transformation in its oxidized products (7-keto and 7β-OH) were measured as markers of the oxidative process. At 140 °C, cholesterol was an oil, and more than 80% of the initial compound disappeared within 1 h of heating, with a significant related increase of the oxysterols 7β-OH and 7-keto, as previously observed [23,24].

**Figure 2:**
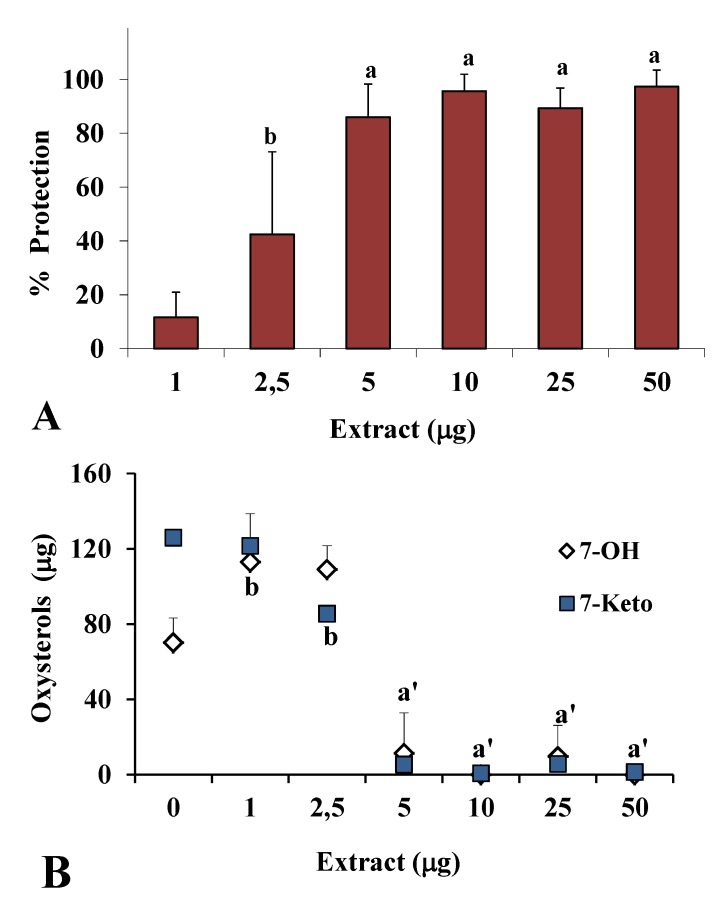
Antioxidant activity (% protection) of different amounts (1–50 μg) of methanolic extract of *C. coccineum* measured during cholesterol degradation at 140 °C for 1 h (**A**) and values (μg) of the oxysterols 7-keto and 7β-OH formed in the absence (0, oxidized control) and in the presence of extract (**B**); a = *P *< 0.001, b = *P *< 0.01 *versus* oxidized control (0), a’ = *P *< 0.001 for both oxysterols *versus* 0 (*n* = 6).

Figure 2 shows the antioxidant activity obtained in the presence of different amounts (1–50 μg) of extract during cholesterol oxidation for 1 h and 2 h. Antioxidant activity is reported as percentage of cholesterol protection (Figure 2A), calculated considering the percentage of sterol consumption in the presence of the antioxidant with respect to total cholesterol consumption without antioxidant (100% of consumption or 0% of protection). The extract showed a significant inhibitory activity of cholesterol degradation from 2.5 μg at the 1 h time point (40% protection) to 5 μg at the 2 h time point (70% protection, data not shown).

Figure 2B shows the values (μg) of the oxysterols 7-keto and 7β-OH formed during cholesterol oxidation at 1 h in the absence (0) or in the presence of methanolic extract. The extract (5 μg) efficiently prevented the formation of the oxysterols at 1 h. 

The scavenging ability of the methanolic extract observed in simple *in vitro *chemical assays was thus confirmed in this model of lipid oxidation. *C. coccineum* extract protected sterol against free radical attack and inhibited oxysterol formation, showing scavenging ability against peroxyl radicals ROO• [21,22]. Cholesterol, present in free and in esterified forms in biological membranes, lipoproteins, and food of animal origin, may undergo oxidation when exposed to oxidative stress, and cholesterol oxidation products, oxysterols, exhibit a host of biological activities of relevance for biomedical research (cytotoxicity, angiotoxicity, and mutagenicity) [23,25].

**Figure 3:**
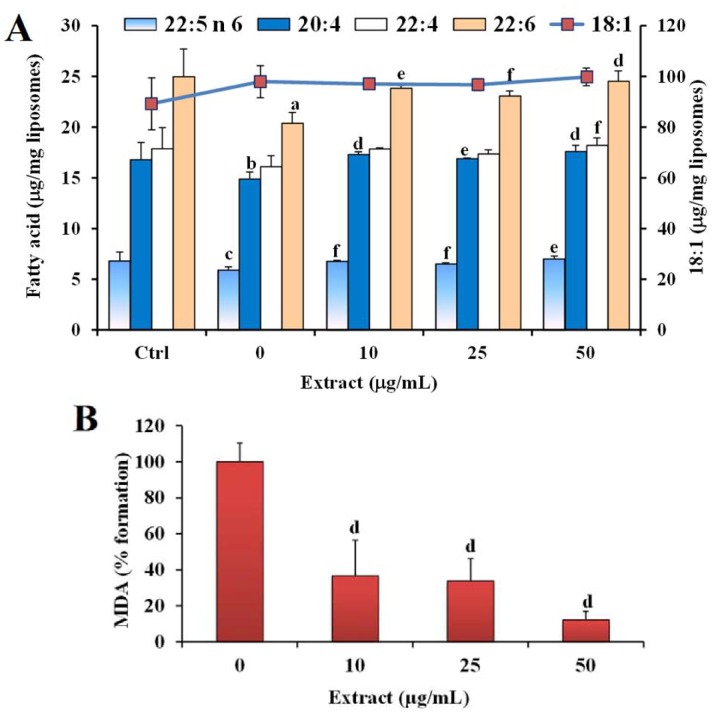
Values of the main unsaturated fatty acids (18:1, 20:4 *n*-6, 22:4 *n*-6, 22:5 *n*-6, and 22:6 *n*-3) expressed as μg/mg liposomes (**A**) and malondialdehyde (MDA) expressed as % of formation (**B**) measured in the control (Ctrl) and during liposome oxidation at 37 °C for 24 h with 5 μM CuSO_4_ in the absence (oxidized control, 0) and in the presence of different amounts (10, 25, 50 μg/mL) of methanolic extract of *C. coccineum*. a = *P* < 0.001, b = *P* < 0.01, c = *P* < 0.05, *versus* Ctrl; d = *P* < 0.001, e = *P* < 0.01, f = *P* < 0.05 *versus* oxidized control; (*n* = 6).

The protective effect of the methanolic extract against the liposome oxidative injury was also evaluated. As an index of the Cu^2+^-induced lipid peroxidation, the variation of the levels of unsaturated fatty acids was analyzed, together with the rise in the level of the oxidation product MDA. Figure 3 shows the values (expressed as μg/mg liposomes) of the main unsaturated fatty acids (18:1, 20:4 *n*-6, 22:4 *n*-6, 22:5 *n*-6, and 22:6 *n*-3) measured in the control (Ctrl) and following liposome oxidation at 37 °C for 24 h with 5 μM CuSO_4_ in the absence (0) and in the presence of different amounts (10, 25, 50 μg/mL) of the extract. 

The Cu^2+^-mediated degradation of liposomes resulted in a significant decrease of the unsaturated fatty acids 22:6 *n*-3, 20:4 *n*-6, and 22:5 *n*-6 in the oxidized control (0) (Figure 3A). The consumption of polyunsaturated fatty acids was accompanied by an increase of MDA level in the system (MDA values: 0.46 ± 0.17 and 3.15 ± 0.60 μM in the Ctrl and oxidized control samples, respectively). The addition of the methanolic extract to the system exerted a significant protection, with respect to the oxidized control, against the oxidation of polyunsaturated fatty acids at all the tested concentrations (Figure 3A), and also significantly reduced the formation of the oxidative product MDA (Figure 3B).

From our observations, the methanolic extract significantly preserved liposome fatty acids from oxidative damage, maybe protecting the lipid particles by scavenging oxygen radicals and chelating coppers ions present at the aqueous phase [24]. Liposomes are considered an important membrane model for the study of lipid peroxidation. Much research has been carried out to understand in depth the effects of lipid oxidation in phospholipid membranes to get tools to prevent, modulate and treat the oxidative damage [26]. The oxidative degradation of unsaturated fatty acids, essential components of biological membranes and LDL, is well known to play a role in the development of tissue damage and in a wide range of pathological events [23,25,26].

## 3. Experimental Section

### 3.1. Chemicals

Unless otherwise specified, all the reagents, standards and solvents were of HPLC graded, purchased from Sigma-Aldrich (Milan, Italy) and used without further purification. CuSO_4_·5H_2_O was supplied by Carlo Erba (Milan, Italy). Standard of cyanidin 3-*O*-glucoside was purchased from Extrasynthese (Genay, France). Ultrapure water (18 mΩ) was obtained with a Milli-Q Advantage A10 System apparatus (Millipore, Milan, Italy).

### 3.2. Plant Materials

*C. coccineum *was collected in south western Sardinia (Italy), in April 2012. Reference material for stems (AR-CC-2012/4/1) was deposited in the collection of the Department of Biomedical Sciences, University of Cagliari. The specimens were kept cool and transferred to the laboratory within one hour from harvest. In a typical collection round, the stems (500 g) were gently cleaned to remove earthy residue and then dried with a blotter. The specimens were cut into slices having a thickness of about 0.5 cm and hung up to dry in air flow at 45 °C for 12 h in a food dehydrator (Rommelsbacher ElektroHausgeräte GmbH, Dinkelsbühl, Germany). After drying, the weight of the dried material was about 185 g. 

### 3.3. Preparation of Extracts

Two steps of extraction were performed on the same plant material. In the first one, dried and ground *C. coccineum* material was extracted with supercritical CO_2_ (SFE) at 40 °C and 250 bars for 4 h. This extraction step, performed in a supercritical laboratory apparatus, produced an apolar fraction (fixed oil). The second extraction step involved the use of a highly polar solvent on the same matrix in order to obtain a polar fraction. To this end, the exhausted *C. coccineum* powder was subjected to maceration by using methanol or water at ambient temperature in a stoppered container with frequent agitation for a period of 72 h at 4 °C. The extracts were filtered and the supernatants obtained were dried in a rotary vacuum evaporator. For HPLC-DAD analysis, an alternative extraction procedure of *C. coccineum* was carried out. Fresh material was extracted with a methanol-water mixture (9:1, v/v). Extraction was performed on whole plant, plant without the external colored layer and on the separated colored layer alone (Figure 1b). For the extraction, 3 g of material were put in a plastic vial with 6 mL of methanol-water mixture, finely ground with an Ultra-Turrax^®^ mixer (International PBI SpA., Milan, Italy) and extracted in an ultrasonic bath for 30 min at 30 °C. The suspension was centrifuged at 6000 rpm, the supernatant was separated and the residue resuspended in 4 mL of methanol and extracted in a sonicator for 30 min. After centrifugation, the supernatant was separated and added to the previous one. The solution was dried *in vacuo* and the residue was kept at −20 °C in the dark until analysis.

### 3.4. Total Phenolics Determination

Folin-Ciocalteu reagent was used to determine total soluble phenolics content according to a previously described method [27]. Gallic acid was used as the standard (linearity range 0.05–0.6 mM), and results were calculated as gallic acid equivalents per gram of dry material (mM GAE/g) using a standard curve.

### 3.5. Total Flavonoids Determination

Total flavonoids content was measured by using a previously described method [10]. Catechin was used as the standard (linearity range 0.1–0.6 mM) and results were expressed as Catechin Equivalent per gram of dry material (mM CE/g).

### 3.6. HPLC-DAD Analysis

Detection and quantitative analyses of phenolic compounds were carried out using an LC-DAD method slightly modified from Tuberoso *et al.* [28]. An HPLC Varian system ProStar was employed, fitted with a pump module 230, an autosampler module 410 with a 10 μL loop, and a ThermoSeparation diode array detector SpectroSystem UV 6000lp (ThermoSeparation, San Jose, CA, USA), set at 280, 360 and 520 nm. Separation was obtained with a Gemini C18 column (150 × 4.60 mm, 3 μm, Phenomenex, Casalecchio di Reno, BO, Italy) using 0.2 M phosphoric acid (solvent A), and acetonitrile (solvent B) at a constant flow rate of 0.8 mL/ min. The gradient (v/v) was generated decreasing from 90% of solvent A to 80% in 10 min; to 60% in 20 min; to 40% in 40 min; to 10% in 50 min. Chromatograms and spectra were elaborated with a ChromQuest V. 2.51 data system (ThermoQuest, Rodano, Milan, Italy). Anthocyanins were detected and dosed at 520 nm, flavonols at 360 nm, and all the other compounds at 280 nm. Gallic acid and cyanidin 3-*O*-glucoside standard solutions were prepared in methanol, and the working standard solutions in ultrapure water. Calibration curves were built with the method of external standard, correlating the area of the peaks with the concentration. The correlation values were 0.9991–0.9999 in the range of 0.2–20 mg/L. Two hundred milligrams of dry extracts were dissolved in 5 mL of the methanol-water mixture (9:1, v/v), diluted with water (1:10–1:50, v/v), and injected in HPLC without any further purification.

### 3.7. DPPH (1,1-Diphenyl-2-picrylhydrazyl Radical) Scavenging Assay

DPPH assay was performed as already described [15]. Trolox was used for the calibration curve (linearity range 5–50 μM), and results were calculated as Trolox Equivalents per gram of dry material (mM TE/g), and as IC_50_ (the extract concentration necessary to decrease DPPH radical concentration by 50%).

### 3.8. Ferric Reducing Antioxidant Power (FRAP)

FRAP was measured as described [15]. Results were calculated as Trolox Equivalents per gram of dry material (mM TE/g) and mmol of Fe(II) per gram of dry material (mmol Fe^II^/g).

### 3.9. Trolox Equivalent Antioxidant Capacity (TEAC) Assay

The TEAC assay was performed according to Re and coworkers [29]. Trolox was used for the calibration curve (linearity range 0.1–0.8 mM) and the results were expressed as Trolox Equivalents per gram of dry material (mM TE/g), and as IC_50_.

### 3.10. ORAC-PYR (Oxygen Radical Absorbance Capacity-Pyrogallol Red) Determination

ORAC-PYR was determined using the pyrogallol red method where APH was the radical releaser [30]. Trolox was used for the calibration curve (linearity range 0.1–0.8 mM), and results were expressed as Trolox Equivalents per gram of dry material (mM TE/g).

### 3.11. Cholesterol Oxidation Assay

The cholesterol oxidation assay was conducted in dry state [23]. Aliquots of 0.5 mL (2586 nmol) of cholesterol solution (2 mg/mL methanol) were dried in a round-bottom test tube *in vacuo* and then incubated in a bath at 140 °C for 1 or 2 h (oxidized controls) under artificial light exposure. Controls (non-oxidized cholesterol) were kept at 0 °C in the dark. In a different set of experiments, aliquots of methanol extract of *C. coccineum* (1–50 μg) in methanol solution (1 mg/mL, 100 μg mL) were added to 0.5 mL of cholesterol solution (2 mg/mL methanol), the cholesterol/extract mixtures were dried *in vacuo* and then incubated in dry state in a bath at 140 °C for 1 or 2 h. During the same time points, non-oxidized control samples of cholesterol were kept at 0 °C in the dark. The oxidation was stopped by cooling and adding 1 mL of methanol. Analyses of cholesterol, 7-ketocholesterol (7-keto), and 7β-hydroxycholesterol (7β-OH) were carried out with a liquid chromatograph (Agilent Technologies 1100, Palo Alto, CA, USA) equipped with a diode array detector (DAD) as previously described [23].

### 3.12. Liposomes Preparation and Oxidation Assays

Liposomes were prepared according to the method of Bangham with slight modifications [24]. Aliquots (300 μg) of liposomes in 1 mL of saline solution were incubated for 24 h in the presence of 5 μM CuSO_4_ at 37 °C in a thermostatic water bath, exposed to air and artificial light. Controls were kept at 0 °C in the dark. Different amounts of methanolic extract (10, 25, 50 μg), redissolved in methanol solution (10 mg/mL, 5 mg/mL and 2 mg/mL), were added to 300 μg of liposomes in 1 mL of saline solution and the mixtures were incubated for 24 h at 37 °C. The oxidation was stopped by cooling the samples in ice/water. Aliquots of liposome samples (900 μL) were immediately dissolved in ethanol and subjected to mild saponification and unsaturated fatty acid analysis by HPLC was conducted as previously described [24]. Aliquots (100 μL) of liposome samples were used for malondialdehyde (MDA) quantification. MDA levels were measured using the method described by Templar and coworkers [31]. MDA-TBA adduct quantification was conducted by HPLC-DAD analysis as previously reported [24].

### 3.13. Statistical Analysis

GraFit 7 (Erithacus Software, London, UK) [32], and R 2.5.1 software (R Foundation for Statistical Computing, Vienna, Austria) [33], Graph Pad INSTAT software (GraphPad software, San Diego, CA, USA) were used to calculate the means and standard deviations. Evaluation of statistical significance of differences was performed by analysis of variance (One-way ANOVA), using Bonferroni Multiple Comparisons Test.

## 4. Conclusions

This study revealed that Maltese mushroom extracts are a valuable source of antioxidants as shown with all the assays used in the present study. Of particular interest is the presence of gallic acid and of cyanidin 3-*O*-glucoside. In recent years, a lot of interest around food supplements enriched with these compounds has developed because of their potential health benefits and their virtual complete lack of harmful side effects. In fact, the number of products present on the market that contain these compounds is rapidly growing. This work extends our understanding of the biological properties of Maltese mushrooms, and paves the path for further investigations, including the possibility of employing this plant as a source of phytochemicals useful in the preparation of nutraceuticals and functional foods, thus re-awakening the ancient traditional uses of *C. coccineum* in the various cultures where this plant has been known for centuries.
